# Choline kinase alpha impairment overcomes TRAIL resistance in ovarian cancer cells

**DOI:** 10.1186/s13046-020-01794-6

**Published:** 2021-01-04

**Authors:** Andrea Rizzo, Alessandro Satta, Giulia Garrone, Adalberto Cavalleri, Alessandra Napoli, Francesco Raspagliesi, Mariangela Figini, Loris De Cecco, Egidio Iorio, Antonella Tomassetti, Delia Mezzanzanica, Marina Bagnoli

**Affiliations:** 1 Department of Research, Molecular Therapies Unit, Fondazione IRCCS Istituto Nazionale dei Tumori, Milan, Italy; 2 Department of Applied Research and Technical Development, Biomarkers Unit, Fondazione IRCCS Istituto Nazionale dei Tumori, Milan, Italy; 3 Department of Research, Epidemiology and prevention Unit, Fondazione IRCCS Istituto Nazionale dei Tumori, Milan, Italy; 4 Present address: UNITECH OMICS Platform, Università degli Studi di Milano, Milan, Italy; 5 Present address: Department of Biomedical and Clinical Sciences “Luigi Sacco”, Università degli Studi di Milano, Milan, Italy; 6 Department of Gynecologic Oncology, Fondazione IRCCS Istituto Nazionale dei Tumori, Milan, Italy; 7 Department of Applied Research and Technological Development, Integrated Biology Platform, Fondazione IRCCS Istituto Nazionale dei Tumori, Milan, Italy; 8 Core Facilities, Istituto Superiore di Sanità, Rome, Italy

**Keywords:** Ovarian cancer, Choline kinase, Metabolic alterations, TRAIL, Apoptosis resistance

## Abstract

**Background:**

Choline kinase-α (ChoKα/*CHKA*) overexpression and hyper-activation sustain altered choline metabolism conferring the *cholinic phenotype* to epithelial ovarian cancer (OC), the most lethal gynecological tumor. We previously proved that *CHKA* down-modulation reduced OC cell aggressiveness and increased sensitivity to in vitro chemotherapeutics’ treatment also affecting intracellular content of one-carbon metabolites. In tumor types other than ovary, methionine decrease was shown to increase sensitivity to tumor necrosis factor-related apoptosis-inducing ligand (TRAIL)-receptor 2 triggering. These effects were suggestive of a potential role for ChoKα in regulating susceptibility to TRAIL cytokine.

**Methods:**

The relationship between ChoKα/*CHKA* and TRAIL-receptor 2 (TRAIL-R2) expression was investigated in silico in OC patients’ GEO datasets and in vitro in a panel of OC cell lines upon transient *CHKA* silencing (siCHKA). The effect of siCHKA on metabolites content was assessed by LC-MS. The triggered apoptotic signalling was studied following soluble-TRAIL or anti-TRAIL-R2 agonist antibody treatment. Lipid rafts were isolated by Triton X-100 fractionation. Preclinical ex vivo studies were performed in OC cells derived from patients’ ascites using autologous PBLs as effectors and a bispecific anti-TRAIL-R2/anti-CD3 antibody as triggering agent.

**Results:**

Here we demonstrate that siCHKA specifically overcomes resistance to TRAIL-mediated apoptosis in OC cells. Upon siCHKA we detected: a significant sensitization to caspase-dependent apoptosis triggered by both soluble TRAIL and anti-TRAIL-R2 agonist antibody, a specific increase of TRAIL-R2 expression and TRAIL-R2 relocation into lipid rafts. In siCHKA-OC cells the acquired TRAIL sensitivity was completely reverted upon recovery of ChoKα expression but, at variance of other tumor cell types, TRAIL sensitivity was not efficiently phenocopied by methionine deprivation. Of note, we were also able to show that siCHKA sensitized tumor cells derived ex vivo from OC patients’ ascites to the cytotoxic activity of autologous lymphocytes redirected by a bispecific anti-TRAIL-R2/anti-CD3 antibody.

**Conclusions:**

Our findings suggest that ChoKα/*CHKA* impairment, by restoring drug-induced or receptor-mediated cell death, could be a suitable therapeutic strategy to be used in combination with chemotherapeutics or immunomodulators to improve OC patients’ outcome.

## Background

In spite of its low occurrence, ovarian cancer (OC) is the primary cause of death among gynecological malignancies [1] mainly due to a late diagnosis and to its propensity to recur as chemoresistant disease with slight improvement of survival rates in recent years [2]*.* Drug resistance is a complex phenomenon associated with several alterations affecting multiple pathways [3]. Cancer cells frequently acquire the ability to resist to apoptotic stimuli induced by chemotherapeutic agents or by death receptors triggering. In this scenario, we contributed to define the mechanisms of OC escape from the death receptor CD95/Fas signaling [4–6]. TNF-related apoptosis-inducing ligand (TRAIL) receptor 2 (R2) is one of the death-inducing receptors triggered by TRAIL, a member of the TNF ligand super-family of cytokines, produced and secreted by most immune cells [7] and considered a promising anticancer agent due to its remarkable ability to selectively kill neoplastic cells sparing normal ones. However, the majority of solid tumors including OC are known to develop resistance to TRAIL-induced apoptosis. Overcoming this resistance still remains an open challenge [8, 9] and in this perspective, we recently showed that bispecific antibodies directed to TRAIL-R2 as tumor-associated antigen (TAA) and to CD3 as triggering molecule were able to redirect polyclonal MHC-unrestricted T lymphocytes cytotoxicity toward OC cells [10].

Among the complex alterations responsible for acquired resistance to death stimuli, there is also the metabolic reprogramming of tumor cells [11]. Interestingly, it was recently reported that methionine deprivation in different cancer models could induce a targetable vulnerability by increasing TRAIL-R2 expression [12–14]. Like many other cancer types, OC shows alterations in the main metabolic pathways [15, 16] and the *cholinic phenotype*, characterized by increased phosphocholine (PCho) intracellular content, is of particular interest [17]*.* We demonstrated that the increase in PCho content in OC cells is mainly maintained by the overexpression and activity of choline kinase alpha (ChoKα*/CHKA*) [18, 19], an enzyme catalyzing choline phosphorylation in the first phosphatidylcholine biosynthetic step [20]. A large body of works indicates that ChoKα is directly associated with malignancy [17, 21] and we contributed to demonstrate its involvement in sustaining the OC transformed status [22]. Indeed, we showed that *CHKA* gene silencing generally hampered OC aggressiveness both in vitro and in vivo by reducing cell proliferation, migration and invasion capabilities [23]. Furthermore, following ChoKα impairment we observed a decreased intracellular content of metabolites other than PCho such as cysteine, serine, methionine and glutathione suggesting deep alterations in circuitry related to one-carbon cycles [24, 25]. In particular we found that the decrease in glutathione level impaired oxidative stress redox status, thus raising intracellular reactive oxygen species that increased in vitro sensitivity to standard chemotherapeutics [24].

Here we aimed to investigate in in vitro and ex vivo OC models whether the ChoKα pro-tumorigenic functions could also affect susceptibility to TRAIL-dependent apoptosis and in particular if the inactivation of ChoKα/*CHKA* expression and function could be a suitable strategy to overcome TRAIL resistance.

## Methods

### Cell cultures

The following OC cell lines were used: SKOV3, OVCAR5, A2780 (obtained from ATCC), INTOV11 (obtained in our laboratory from a high-grade serous OC [23]), A2774 (provided from dr. S. Ferrini, IRCCS Ospedale Policlinico S. Martino, Italy) were maintained in RPMI1640 (Sigma-Aldrich) supplemented with 10% FCS (Lonza) and 2 mM L-glutamine (Lonza); OVCAR3 (from ATCC) were maintained in RPMI1640 supplemented with 20% FCS, 2 mM L-glutamine, 10 mM HEPES, 1 mM Na pyruvate; OAW42 (provided by dr. A. Ullrich, Max-Planck Institute, Germany) were maintained in EMEM (Sigma-Aldrich) supplemented with 10% FCS, 2 mM L-glutamine and non essential amino acids (Lonza). Cells were cultured at 37 °C in a humidified incubator under 5% CO_2_ and genotyped at Functional Genomic facility of our Institute, using Stem Elite ID System (Promega) according to ATCC guidelines. Cells were routinely confirmed to be mycoplasma-free by MycoAlert Mycoplasma Detection Kit (Lonza). For methionine deprivation experiments, RPMI1640 L-methionine free medium (Thermo-Fisher) was used.

### Transient gene silencing

A total of 1.8 × 10^5^ OC cells/well were seeded on six-well plates (Eppendorf) and transfected with a final concentration of 40 nM small interfering RNA (Supplementary Table 1). Transfection was carried using Lipofectamine2000 (ThermoFisher). The efficacies of silencing and related biological effects were assessed 72 h after transfection. Recovery of ChoKα expression was evaluated 144 h after transfection.

### Quantitative real time PCR

Total RNA extraction and qRT-PCR were performed as previously described [23]. The ΔCT method was used to determine the quantity of the target sequences and *GAPDH* or *RPL13A* were amplified as housekeeping controls. For probes used see Supplementary Table 1.

### Western blotting

Cells were lysed using RIPA buffer [19] while ex vivo derived samples were directly lysed with NuPAGE LDS buffer (ThermoFisher). Proteins solving, immunodecorations, blots acquisition and densitometric analysis were performed as previously described [23]. Antibodies (Supplementary Table 1) were diluted as recommended.

### Metabolomic profiling

For metabolites analysis 2 × 10^5^ cells were lysed in 500 μl H_2_O + 0.1% formic acid and centrifuged at 3.500 rpm for 15 min in Amicon Ultra-0.5 membrane tubes. Elutions (10 μl) were injected in triplicates and analyzed using the following platforms: I) Accela LC systems (Thermo Scientific) connected to a TSQ Quantum Access Mass Spectrometer (Thermo Scientific) equipped with a HESI ion source separating the analytes at 35 °C on a XBridge BEH300 C18 column (3.5 μm, 2.1 × 150 mm; Waters); II) ExionLC AD system (SCIEX) connected to a TripleTOF 6600 System (SCIEX) equipped with a Turbo V ion source with ESI Probe separating analytes at 40 °C with CORTECS UPLC T3 column (1.6 μm, 2.1 × 150 mm; Waters). Run was performed in isocratic mode at 97% mobile phase A (0.1% formic acid in water) and 3% mobile phase B (0.1% formic acid in methanol) in 5 min at a flow rate of 300 μl/min. Mass spectra were collected in positive ion mode. Selected Reaction Monitoring transition of the precursor to product ions was used for quantification. Individual stock solutions of methionine and phosphocholine were prepared from separate weightings of each compound dissolved in 0.1 M HCl. A standard mix with all analytes at 15, 62, 250 and 1000 ng/ml concentrations for calibration curves. Deuterates form of the two metabolites (Met-d2 and PCho-d9, Merck Millipore) at 10 μg/ml were used as internal standards. For each curve, the absolute peak-area ratios of standard to the internal standard were calculated and plotted against the nominal concentration. Calibration curves were generated by weighted (1/x) linear regression analysis. To account for bias in sensitivity of metabolites detection of the two different platforms, data were reported as relative ratio of metabolite content between siCHKA vs control cells.

### Flow cytometry

For detection of TRAIL-Rs membrane expression, cells were incubated with primary antibodies for 45 min on ice (or at 37 °C where indicated). For detection of total TRAIL-R2 expression, cells were permeabilized with eBioscience Permeabilization Buffer (Invitrogen) before staining. Antibodies (Supplementary Table 1) were diluted as recommended. Fluorescence index was calculated as ratio between mean fluorescence intensity values of TRAIL-Rs and negative controls (IgG isotype PE-conjugated or ALEXA488 in direct or indirect immunostaining, respectively).

Quantification of apoptosis was carried out by staining cells with FITC-conjugated Annexin V (Abcam) and To-PRO-III cyanine dye (Invitrogen). Mitochondrial membrane potential (ψ_m_) was determined by staining cells with 5 μg/ml JC-1 dye (Invitrogen) for 10 min at 37 °C.

Fluorescence labeling was measured acquiring samples by the LSRFortessa system (Becton-Dickinson), data were analyzed and processed with the FlowJo software (Tree Star Inc).

### Cell growth inhibition assay

Cells seeded in triplicates at 10^4^/well in 96-well plates (Costar) were treated for 16 h with 0.5 μg/ml sTRAIL (Killer TRAIL soluble human recombinant, Enzo LifeSciences) or 25 μg/ml specific agonist anti-TRAIL-R2 antibody [10]. For caspases inhibition, cells were incubated with 50 μM of pan-caspases, Caspase-9 or Caspase-8 inhibitors (Z-VAD-FMK, Z-LEHD-FMK, Z-IETD-FMK, Calbiochem) for 1 h before sTRAIL exposition. For each test, untreated triplicates were seeded as control. Growth inhibition was assessed using the CellTiter-GLO luminescent cell viability assay (Promega) measuring luminescence by an Ultra reader apparatus (Tecan Group).

### Public datasets analysis

Expression of *CHKA* and *TNFRSF10B* were explored in public available gene expression studies on GEO repository (http://www.ncbi.nlm.nih.gov/geo) reporting data for ovarian surface epithelium (OSE), low malignant potential (LMP), low/high grade tumors profiled on the same microarray platform (Affymetrix U133 plus 2.0). Nine datasets were selected: GSE18520, GSE27651, GSE14001, GSE12172, GSE14407, GSE23391, GSE29450, GSE20565, GSE19352 accounting for a total of 333 samples including 45 OSE; 38 LMP; 57 LG; 191 HG tumors. Raw data were retrieved and signal intensity was normalized within each individual dataset using Robust Multi-Array Average [26]. The datasets were integrated following a meta-analysis approach by applying analytical methods for data normalization and batch effect correction as previously described [27]. The log2 expression of *CHKA* and *TNFRSF10B* identified by 204266_s_at and 209295_at Affymetrix probe sets, respectively, were retrieved. Boxplots were generated using the “ggplot2” R package with the notched boxplot function. The *p*-values were calculated by the Kruskal–Wallis test. Correlation plot using scatter graph between *CHKA* and *TNFRSF10B* is visualized by “ggpubr” R package and correlation assessed by Pearson’s test.

### Triton X-100 soluble and insoluble fractionation

Separation of TX-soluble and TX-insoluble fractions was performed as already described [28]. Proteins of TX-soluble and an equivalent volume of TX-insoluble fractions were analyzed by Western blotting. Cav-1 or Flot-1 were used as markers of the TX-insoluble fraction.

### Patients’ derived samples and lymphocytes retargeting

Cells from the ascitic fluid of a chemo-naive stage IIIC patient (TEM1510A) undergoing surgery for a high grade serous OC were collected by centrifugation and seeded in a flask for 30 min. Non-adherent tumor cells clumps were recovered and maintained in RPMI1640 in according to cell culture conditions until processed for further analysis. Autologous peripheral blood lymphocytes (PBLs) were collected as described [10]. Patients had signed an informed consent form, in accordance with the institutional ethics committee guidelines and following the principles of the Declaration of Helsinki. For PBLs retargeting experiment, growth inhibition was evaluated by MTT assay [10] using PBLs as effectors, tumor cells isolated from OC patients’ ascitic fluids as targets and the TRAIL-R2 × CD3 bispecific antibody (bsAb) as retargeting agent [29].

### Microscopy

Cells were analyzed using an Eclipse TE2000-S microscope with a 20× or 40× 0.75NA objectives (Nikon). Images were acquired with ACT-1 software (Nikon) and processed using ImageJ and Adobe Photoshop softwares.

### Statistical analysis

Data were analyzed using Prism GraphPad software. Statistical significance of differences was determined by Student’s *t*-test, as specified. Asterisks in all figures denote a statistically significant difference in comparison with the relative control *P < 0.05; **P < 0.01; ***P < 0.001. Data reported are the mean ± S.D. of at least three independent experiments unless otherwise indicated.

## R**esults**

### *CHKA* silencing overcomes TRAIL resistance

**Fig. 1:**
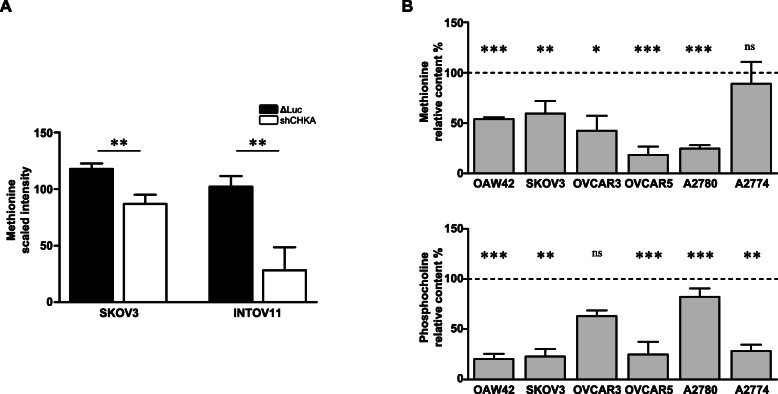
*CHKA* silencing affected methionine intracellular content. **a.** Quantification of methionine signals in control (ΔLuc) and *CHKA* stably silenced (shCHKA) OC cell lines obtained by mass spectrometry with the Metabolon Technology [24]. **b.** Content of methionine (*upper panel*) and phosphocholine (*lower panel*) obtained by mass spectrometry analysis of OC cell lines; data are reported as percentage of transient siCHKA/siNT ratio. Asterisks are referred to statistically significant differences

The deprivation of methionine, a key metabolite of one-carbon metabolism [30], was recently shown to induce an increased vulnerability to TRAIL-R2-dependent apoptotic signaling in different in vitro cancer models [12--14]. Interestingly, along with reduced aggressiveness and increased sensitivity to chemotherapeutics, the metabolic profiles of two *CHKA* stably silenced OC cell lines showed the perturbation of several metabolites related to one-carbon cycles [24]. Notably, in these *CHKA* stably silenced models, methionine content was significantly decreased as compared to their relative controls (Fig. 1a). Taken together, these observations suggested that interfering with ChoKα-dependent phenotype could ultimately increase TRAIL sensitivity in OC cells.

To support these findings, a broad panel of OC cell lines representative of different OC subtypes, including those already characterized for *cholinic phenotype* [24], were analyzed for methionine content following transient *CHKA* silencing (siCHKA, Fig. S1). The decrease in methionine was validated by mass spectrometry (LC-MS) in all the models tested together with the reduction of PCho as metabolic readout of efficacy of siCHKA (Fig. 1b).

**Fig. 2:**
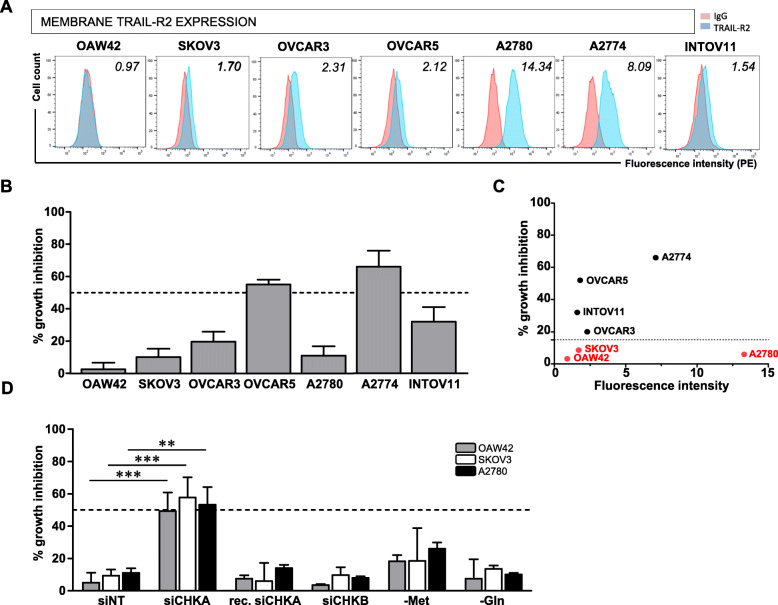
TRAIL resistance in OC cell lines is not related to basal TRAIL-R2 expression levels and is specifically overcome by siCHKA. **a.** Representative flow cytometry analysis showing surface membrane expression of TRAIL-R2 in wild type OC cell lines. Fluorescence index is reported. **b.** Percentage of TRAIL-mediated growth inhibition of wild type OC cell lines following 16 h treatment with sTRAIL as compared to relative untreated cells. **c.** Correlative plot of TRAIL-R2 membrane basal expression (fluorescence index) versus percentage of TRAIL-mediated cell growth inhibition. In red are indicated TRAIL-resistant cell lines. **d.** OC cells sensitivity to sTRAIL treatment following: siCHKA; recovery of ChoKα expression (144 h after silencing); siCHKB; deprivation of methionine (−Met) and deprivation of glutamine (−Gln). Asterisks are referred to statistically significant differences. Dotted lines indicated 50% growth inhibition

To verify the hypothesis that ChoKα/*CHKA* impairment could ultimately affect activation of TRAIL-R2-dependent apoptotic signaling, we characterized the basal membrane expression of TRAIL-R2 and the sensitivity to TRAIL cytokine (sTRAIL) treatment in all OC in vitro models. Flow cytometry analysis showed that TRAIL-R2 membrane expression was globally low in all the cell lines tested except for A2780 and A2774 cells (Fig. 2a). Growth inhibition assay indicated low cell sensitivity to TRAIL, growth inhibition exceeded 50% in only two out of seven OC lines (Fig. 2b). In line with literature data [31], no direct correlation (*P* = 0.87) was observed between basal expression of TRAIL-R2 and TRAIL sensitivity (Fig. 2c).

To characterize the effects of siCHKA on TRAIL-triggered cell death we specifically focused on the three frankly resistant OC cell lines never exceeding 20% growth inhibition following sTRAIL exposure: OAW42, SKOV3 and A2780. Following siCHKA we detected a significant increase of TRAIL sensitivity in all three cellular models compared to their relative controls (siNT) (Fig. 2d). Notably, following recovery of ChoKα expression after transient silencing (Fig. S2A), the sensitivity to sTRAIL treatment was completely reverted dropping at level of control cells (Fig. 2d). Moreover, cells sensitization was specifically dependent upon siCHKA since by silencing the beta isoform (ChoKβ/*CHKB*) of Choline kinase (siCHKB, Fig. S2B) TRAIL-mediated cell death did not increase (Fig. 2d). To determine whether the effects of siCHKA on TRAIL sensitization could be directly dependent to the observed methionine decrease (Fig. 1b, *upper panel*), the three OC cell lines were grown in standard or methionine-free media for 72 h. At variance with results described in other tumor models [12--14], we observed only a moderate effect of methionine deprivation on TRAIL sensitization. Furthermore, such effect was similar to that observed following deprivation of glutamine, an essential aminoacid not directly related to methionine and/or choline metabolisms (Fig. 2d).

Taken together these results confirmed that siCHKA was specifically capable to overcome TRAIL resistance in OC cells but this effect was only partially affected by metabolic alterations related to methionine deprivation.

### sTRAIL triggers an efficient apoptotic cascade in siCHKA OC cells

**Fig. 3:**
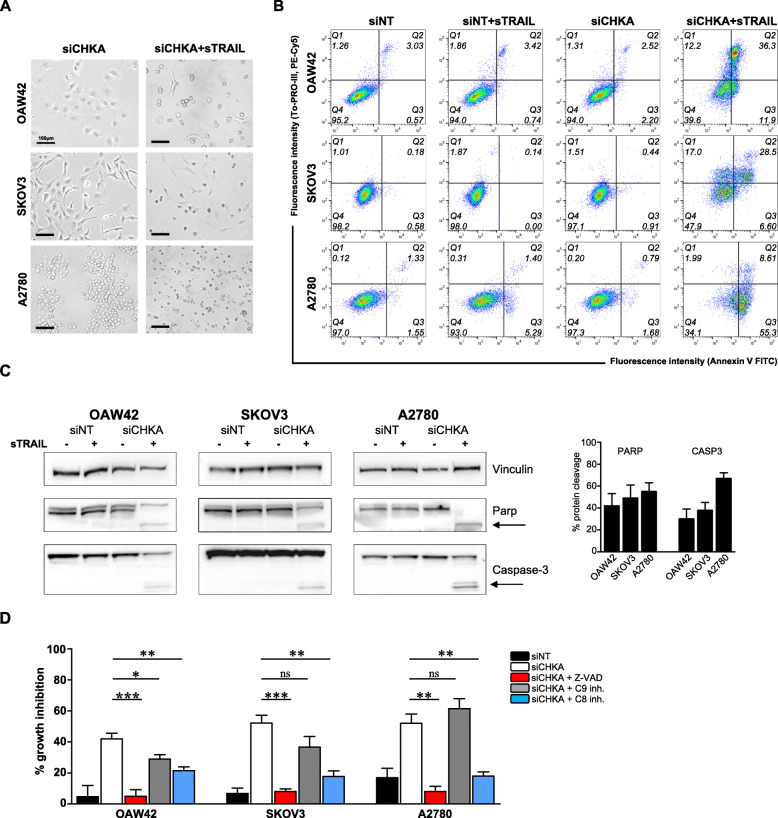
sTRAIL treatment triggered an efficient apoptotic cascade in siCHKA OC cells. **a.** Live 40× microscopy images of siCHKA OC cell lines untreated or treated with sTRAIL. Scaled bar = 100 μm. **b.** Representative flow cytometry analysis showing Annexin V/To-PRO-III staining of siCHKA and control OC cells following sTRAIL treatment. Q4: healthy cells, Q3: early apoptotic cells, Q2: late apoptotic cells, Q1: necrotic cells/debris (for each quadrant, the cell percentage on total count events is reported). **c.** *Left panel*: Representative Western blotting for PARP and Caspase-3 expression in siCHKA and siNT cell lines after sTRAIL treatment. Arrows indicate the cleaved forms of the proteins. Vinculin was used as loading control. *Right panel:* densitometric analysis of cleaved PARP and Caspase-3 proteins in siCHKA sTRAIL-treated cells versus control (untreated) cells. **d.** Percentage of TRAIL-mediated cell growth inhibition (16 h sTRAIL treatment) of OC cells siNT (black bars) and siCHKA (white bars) alone or in presence of pan-caspases inhibitor (red bars), Caspase-9 inhibitor (grey bars), Caspase-8 inhibitor (blue bars). Statistical differences are reported

Starting from 2 h of incubation, sTRAIL treatment promoted in siCHKA cells an enhanced cell shrinkage followed by a general cell detachment from the culture plate (Fig. 3a), suggesting an apoptotic process. Indeed, the early apoptotic event of Annexin V binding on cells’ membrane was evident in all three OC cell lines upon siCHKA and sTRAIL treatment (Fig. 3b). In addition, while sTRAIL treatment of control cells did not affect PARP integrity, its cleavage was evident in treated siCHKA cells thus indicating an efficient apoptotic cascade. Furthermore, the activation of the final effector Caspase-3 was also evident as indicated by the presence of its cleaved form (Fig. 3c).

The general involvement of caspases in TRAIL-mediated cell death following siCHKA was confirmed by pre-treating cells with the pan-caspase inhibitor Z-VAD that completely abolished TRAIL sensitivity in all siCHKA OC models (Fig. 3d, red bars). To further analyze whether extrinsic and intrinsic apoptotic pathways differently contributed to the execution of cell death program, before sTRAIL exposure we incubated siCHKA and control cells to specific inhibitors of initiator Caspase-9 or initiator Caspase-8 (mainly involved in intrinsic or extrinsic pathways respectively). Inhibition of Caspase-9 slightly affected apoptosis only in siCHKA OAW42 and SKOV3 cell lines, suggesting that the involvement of intrinsic pathway has different relevance in the different models (Fig. 3d, grey bars). Furthermore, in spite of a mitochondrial membrane potential decrease observed following siCHKA (Fig. S3A), we did not detect the truncated form of the mitochondrial transducer Bid nor altered expression of the pro- and anti-apoptotic molecules affecting mitochondrial function such as Bcl-2 and XIAP (Fig. S3B) substantially in agreement with the weak effects observed following Caspase-9 inhibitor treatment.

At variance, the specific inhibition of Caspase-8 significantly affected apoptosis in all the cell lines tested thus indicating that the main contribution to execution of the apoptotic program is given by activation of the extrinsic pathway (Fig. 3d, blue bars).

### Sensitization to sTRAIL treatment of siCHKA OC cells is specifically dependent on TRAIL-R2 membrane expression and triggering

In order to check the molecular mechanisms involved in the siCHKA-dependent OC cells sensitization to apoptosis, we analyzed the effect of siCHKA on TRAIL-R2 transcript (*TNFRSF10B)* and protein expression.

**Fig. 4:**
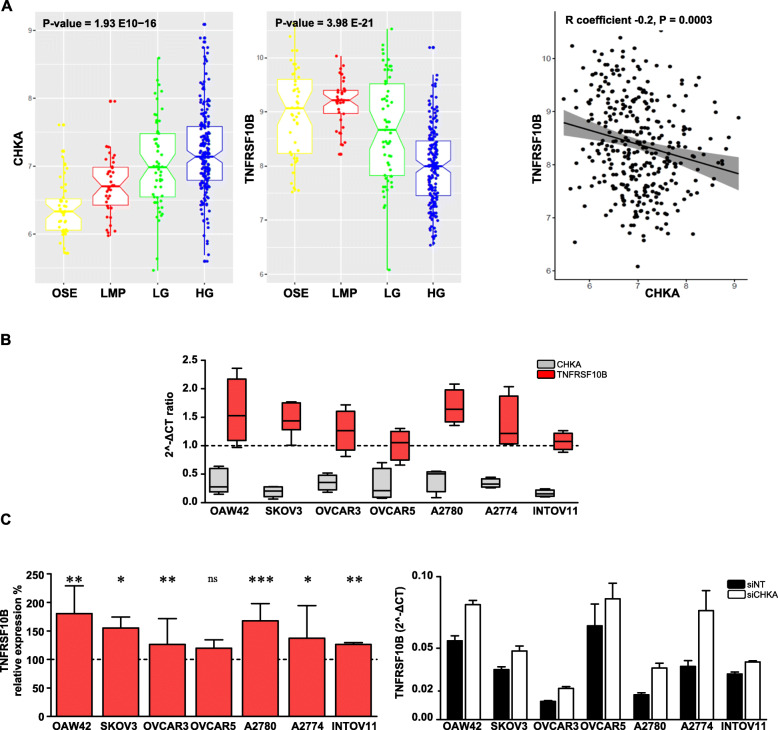
*CHKA* and *TNFRSF10B* expression are inversely related in OC samples and cell lines. **a.** *Left panels:* Notched box plots showing the log2 expression values of *CHKA* and *TNFRSF10B* within preparations of normal ovarian surface epithelium (OSE), low malignant potential (LMP), low grade (LG), high grade (HG) tumors. The boxes represent the interquartile range; the notches the 95% confidence intervals of the median; the whiskers the ranges within 1.5× interquartile range of the upper or lower quartile. *Right panel:* Inverse correlation of log2 expression between *CHKA* and *TNFRSF10B* in the meta-analysis, 95% confidence area (grey region). **b.** Comparative analysis of *CHKA* (red boxes) and *TNSFRSF10B* (grey boxes) transcripts variation in OC cell lines following siCHKA. For each transcript, the relative change as compared to siNT cells is shown. Dotted line (=1) represents no transcript expression variation between siCHKA and siNT samples. **c.** Relative quantification of *TNFRSF10B* transcript expression reported as percentage of 2^-ΔCT siCHKA/siNT ratio (*left panel*); asterisks are referred to a statistically significant differences; a representative experiment is shown (*right panel*)

In the previously described *CHKA* stably silenced OC model [23] we detected a 1.27 fold increase (*P* = 0.0066, FDR = 0.2) of the *TNFRSF10B* gene compared to controls [23]. Interestingly, by checking the expression of *CHKA* and *TNFRSF10B* in nine public available datasets reporting data for ovarian surface epithelium (OSE), low malignant potential (LMP), low/high grade tumors for a total of 333 samples (Methods), we observed a progressive increase of *CHKA* along with malignancy, thus sustaining our previously published data [18, 19], and a corresponding decrease of *TNFRSF10B* (Fig. 4a, *left panels*). Expression of the two genes appeared anti-correlated (Fig. 4a, *right panel*). Notably, also in the panel of *CHKA*-silenced OC cell lines we detected an increase, although moderate, of *TNFRSF10B* transcript expression compared to controls (Fig. 4b and c).

**Fig. 5:**
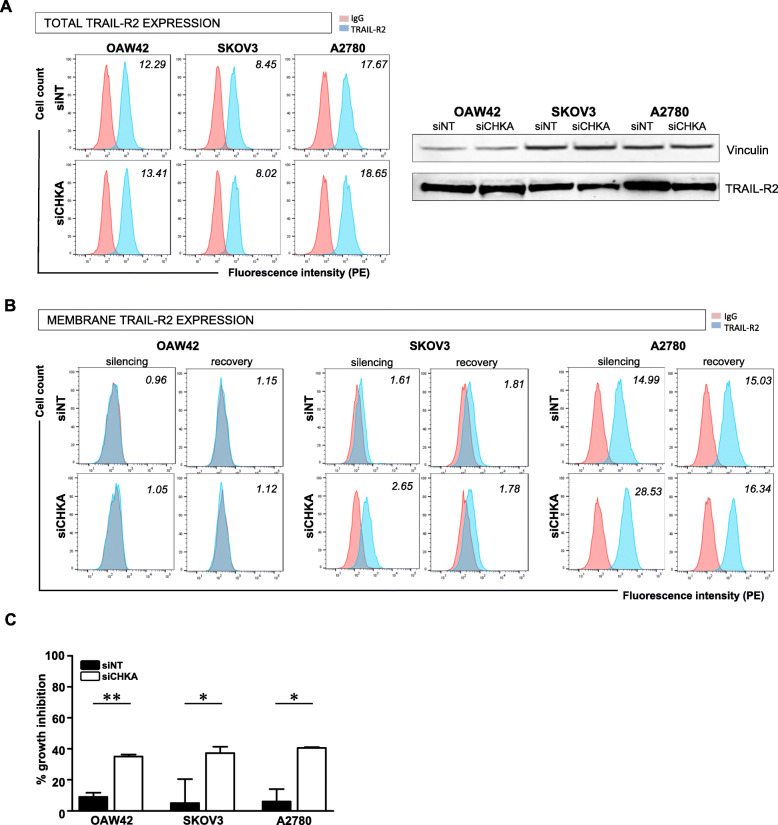
*CHKA* silencing increased TRAIL-R2 membrane expression and triggering/function. **a.** Expression of total TRAIL-R2 protein assessed by both flow cytometry after cells permeabilization (*left panel*) and Western blotting (*right panel*) in siCHKA and siNT cell lines. **b.** Membrane surface expression of TRAIL-R2 detected by flow cytometry after *CHKA* silencing and recovery of ChoKα expression. In both A and B, representative experiments are shown. Fluorescence index are reported. **C.** Percentage of growth inhibition obtained following 16 h treatment with specific anti-TRAIL-R2 agonist antibody; asterisks are referred to statistically significant differences

We thus checked TRAIL-R2 protein expression in the three selected TRAIL-resistant siCHKA OC models. Although no appreciable variations of total protein amount were detectable (Fig. 5a), we clearly observed by flow cytometry an increase of TRAIL-R2 membrane exposure in siCHKA SKOV3 and A2780 while no changes were evident in siCHKA OAW42 cells (Fig. 5b). Interestingly, in SKOV3 and A2780 models, the basal TRAIL-R2 membrane level was completely rescued upon the natural recovery of ChoKα expression after silencing (Fig. 5b) in accordance with the reversion of sensitivity to sTRAIL treatment previously observed (Fig. 2d). Furthermore, no effects on TRAIL-R2 membrane exposure were detected following siCHKB (Fig. S4A).

In order to assess whether the observed sensitization to apoptosis was specifically related to TRAIL-R2 upregulation we checked gene and membrane expression of the other TRAIL-R family members. While a global increase of *TNFRSF10B* was evident in all siCHKA cells, different variations without univocal trend in transcript expression were observed for *TNFRSF10A*/TRAIL-R1, *TNFRSF10C/*TRAIL-R3 and *TNFRSF10/*TRAIL-R4 (Fig. S4B). Parallel flow cytometry analysis showed no evident alteration of TRAIL-R1, R3, R4 membrane expression in siCHKA cells compared to controls (Fig. S4C). Most importantly, the specific involvement of TRAIL-R2 in sensitization to apoptosis following siCHKA was confirmed by using a specific anti-TRAIL-R2 agonist antibody [10] (Fig. 5c).

### *CHKA* silencing promotes TRAIL-R2 relocation in functional membrane microdomains

**Fig. 6:**
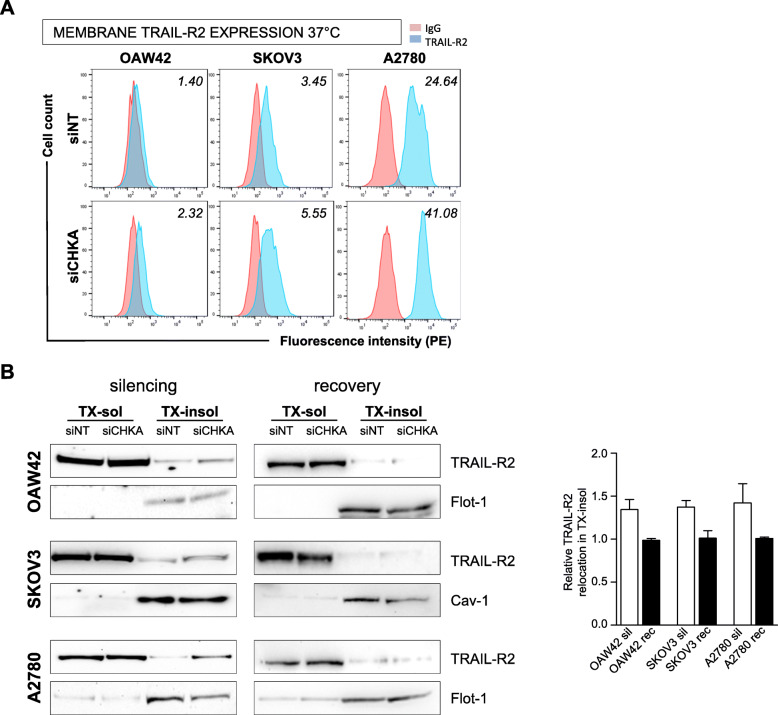
*CHKA* silencing promoted TRAIL-R2 membrane relocation. **a.** Representative flow cytometry analysis indicating TRAIL-R2 membrane expression following primary antibody incubation at 37 °C in siCHKA and control OC cell lines. Fluorescence index are reported. **b.** Representative Western blotting analysis of TX-soluble and TX-insoluble fractions for TRAIL-R2 expression after *CHKA* silencing (*left panel)* and following recovery of ChoKα expression (*middle panel*): Cav-1 and Flot-1 were used as markers of TX-insoluble fractions. *Right panel*: densitometric analysis of relative TRAIL-R2 relocation in TX-insoluble fraction following silencing (white bars) and recovery of Chokα (black bars)

Notably, the effects of treatment with both anti-TRAIL-R2 agonist antibody and sTRAIL were evident also in siCHKA OAW42 (Fig. 2d) that, apparently, failed to up-regulate TRAIL-R2 membrane expression (Fig. 5b). We thus hypothesized that mechanisms inherent to membrane architecture and plasticity could be involved. Flow cytometry analysis for TRAIL-R2 expression was therefore performed at 37° rather than 4 °C in order to mimic the physiological conditions of cell culturing. With this approach an increase of TRAIL-R2 membrane expression (but not of any other member of TRAIL, Fig. S4D) was evident also on siCHKA OAW42 cells (Fig. 6a).

A possible TRAIL-R2 relocation in specific membrane microdomains may promote an efficient cell death signaling process [32]. Accordingly, we verified that following siCHKA there was an increase (from 1,3 to 1,5 fold) in the relative amount of TRAIL-R2 in the Triton X-100-insoluble fraction of lysates corresponding to lipid rafts (Fig. 6b, *left panel*). Interestingly, following the recovery of Chokα expression after transient silencing (Fig. S2A), no appreciable differences in the relative TRAIL-R2 distribution in Triton X-100-insoluble fraction were detected between siCHKA-recovered and control cells (Fig. 6b, *middle panel*).

### TRAIL-R2 increase induced by siCHKA may be a suitable TAA for lymphocyte retargeting

**Fig. 7:**
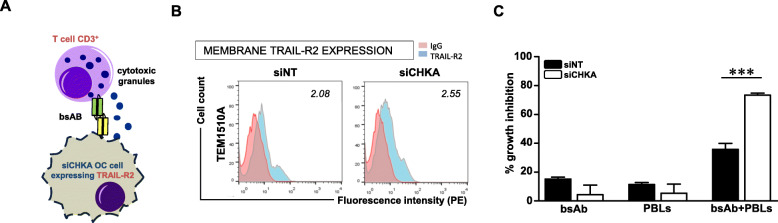
TRAIL-R2 induced by siCHKA as TAA for retargeting of lymphocyte cytotoxicity by anti-TRAIL-R2/anti-CD3 bispecific antibody. **a.** Cartoon depicting the retargeting of lymphocyte against siCHKA ex vivo OC cells expressing TRAIL-R2, by anti-TRAIL-R2/anti-CD3 bispecific antibody (bsAb). **b.** Representative flow cytometry analysis indicating TRAIL-R2 membrane expression in siCHKA and siNT ex vivo patient-derived ascitic tumor cells. Fluorescence index are reported. **c.** Percentage of growth inhibition of patients-derived ex vivo siCHKA and control OC cells in the presence of anti-TRAIL-R2/anti-CD3 bsAb, lymphocytes alone (PBLs) or their combination; asterisks are referred to a statistically significant difference

Since we recently showed [29] that a bispecific antibody (bsAb) directed to TRAIL-R2 as TAA and to CD3 as triggering molecule, can efficiently redirect CD3^+^ T lymphocytes cytotoxicity toward cancer cells (Fig. 7a), we explored if the increase of TRAIL-R2 following siCHKA could also result in an improvement of the bsAb-mediated T cell anticancer activity. To obtain a proof of principle of a clinically relevant application of this effect, we tested the ability of the bsAb to retarget the cytotoxic activity of autologous lymphocytes against ex vivo OC cells derived from patients’ ascitic fluid silenced for *CHKA* (Fig. S5A). Also in this ex vivo cellular model, siCHKA induced a consistent increase of TRAIL-R2 membrane exposure (Fig. 7b) thus allowing sensitization of patients-derived cancer cells to bsAb-mediated lymphocyte retargeting with a significant increase of growth inhibition (Fig. 7c).

## Discussion

We previously contributed to demonstrate that ChoKα/*CHKA* is involved in the maintenance of OC aggressiveness and drug resistance [23, 24]. Here we show that *CHKA* silencing (siCHKA), by increasing TRAIL-R2 membrane expression and availability interferes also with OC cells susceptibility to receptor-mediated apoptosis efficiently overcoming TRAIL resistance in vitro and redirecting ex vivo lymphocytes cytotoxicity triggered by bsAb to TRAIL-R2/CD3.

*TNFRSF10B/*TRAIL-R2 is a TNF-related membrane receptor whose triggering by its ligand (TRAIL) transmits the apoptotic signal in cancer cells sparing normal cells [33, 34]. This peculiar characteristic opened a therapeutic window for the development of new cancer therapeutics [35, 36]. However, despite promising in vitro and preclinical results demonstrating successful antitumor activity with low side effects, lack of a therapeutic outcome has been observed in clinical trials [37]. The major limitation to TRAIL-targeted therapy is the acquisition of apoptosis escape mechanisms that prevent TRAIL-mediated killing of tumor cells [38] including OC [9].

Reasonably, a big effort is devoted to search for appropriate combinatorial treatments to overcome resistance effects [39] and vulnerability of tumor cells to specific amminoacid deficiency [40, 41] could offer new targeting approaches. In particular, methionine restriction has been shown to enhance efficacy of anti-tumor cytotoxic agents [42] or apoptotic response [43] and interestingly, the upregulation of TRAIL-R2 was described in different tumor models [12--14]. We have already evidence that in OC cell lines *CHKA* silencing perturbed metabolic circuitry related to one-carbon cycles [24, 25] and our current data showed a decrease in methionine together with an increase in *TNFRSF10B* in siCHKA cells. A significant inverse correlation between *CHKA* and *TNFRSF10B* expression was observed in the in silico analysis of more than 300 OC samples at different grade of malignancy*.* Consistent with our previous data [19], *CHKA* expression increased along with malignancy while a corresponding decrease of *TNFRSF10B* was evident, thus supporting the notion that the *cholinic phenotype* might interfere with TRAIL receptors expression. In OC models the sensitization to TRAIL-dependent apoptosis mainly relied on ChoKα-related expression and functions rather than on methionine deprivation. Indeed, while the recovery of ChoKα expression in siCHKA cells caused the complete reversion to the resistant phenotype, methionine deprivation had only a minor effect on TRAIL-sensitivity. In addition, OC sensitivity to TRAIL-mediated apoptosis was not modified by siCHKB further supporting previous findings showing that harnessing of OC aggressiveness is a peculiar characteristic of siCHKA [23, 24].

In all three cellular models selected as TRAIL resistant, siCHKA-dependent sensitization to TRAIL-mediated cell death relied on activation of extrinsic apoptotic pathway as demonstrated by the reversion of apoptosis by Caspase-8 specific inhibition. Furthermore, apoptosis appears to be exclusively related to TRAIL-R2 membrane availability since we did not observed a consistent up-modulation of TRAIL-R1 or down-modulation of the decoy receptors TRAIL-R3 and R4. We also demonstrated the effectiveness of siCHKA in up-modulating TRAIL-R2 ex vivo in tumor cells derived from OC patients’ ascitic fluids. Late stages OC present with abundant malignant ascites rich of cellular components (tumor and non tumor cells) and pro-tumor soluble factors [44, 45] able to attenuate TRAIL-induced apoptosis and counterattack apoptotic signaling [46, 47]. By using a bsAb directed against TRAIL-R2 as TAA and the T cell receptor associated CD3 as triggering molecule, we were able to induce the killing of cancer cells grown in a pro-tumor microenvironment using autologous lymphocytes mimicking a real human in vivo situation [10, 29].

The majority of OC cell lines selected for our analyses were TRAIL resistant without a correlation with basal TRAIL-R2 expression level. Notably in the siCHKA OAW42 cell line that clearly acquired a TRAIL-sensitive phenotype, TRAIL-R2 membrane expression became evident only following incubation at 37 °C suggesting the involvement of membrane plasticity. Moreover, we observed in all the siCHKA cell lines a general increase of the relative amount of TRAIL-R2 located in specific membrane microdomains correspondent to lipid rafts [32] and the loss of TRAIL-R2 relocation following recovery of ChoKα expression. In these microdomains, which are known regulatory platform of membrane-associated signaling molecules since proteins have limited ability to diffuse over plasma membrane, protein-protein interactions are facilitated and death receptors signaling is favored [32]. These observations, in line with the pivotal role of ChoKα in phosphatidylcholine biosynthesis, suggested that ChoKα impairment could possibly modulate phospholipids homeostasis thus affecting overall membranes architecture and properties (plasticity). This phenomenon could favor TRAIL-R2 relocation into lipid rafts facilitating death receptor triggering and execution of the apoptotic signaling.

## Conclusions

We demonstrated the involvement of the *cholinic phenotype* in regulation of death receptors expression and overall susceptibility of OC cell to their triggering. Our data by confirming previous observation obtained in a colorectal cancer model [48], overall foster the potential usefulness of ChoKα targeting in a clinical setting.

ChoK-α impairment, by simultaneously interfering with multiple mechanisms on which tumor cells rely for proliferation/survival, would reduce the fitness of tumor cells making them more vulnerable to external stress. The consequential weakness is expected to ultimately reduce the ability of OC cells to resist to drug and/or cytokine treatments.

## Supplementary Information

**Additional file 1: Supplementary table 1** Full list of antibodies, probes and siRNA used.

**Additional file 2: Supplementary Figures 1-5.**

## Data Availability

All data generated or analyzed during in this study are included in this published article.

## Abbreviations

bsAb: Bispecific antibody
ChoKα: Choline Kinase alpha
GEO: Gene Expression Omnibus repository
LC-MS: Liquid chromatography–mass spectrometry
LMP: Low malignant potential
OC: Ovarian cancer
OSE: Ovarian surface epithelium
PBLs: Periferal blood lymphocytes
PCho: Phosphocholine
siCHKA: *CHKA* silencing
siCHKB: *CHKB* silencing
siNT: No-target silencing
sTRAIL: soluble TRAIL
TAA: Tumor associate antigen
TNF: Tumor necrosis factor
TRAIL: TNF-related apoptosis-inducing ligand
TRAIL-Rs: TRAIL receptors
TX: Triton X-100
