# Supplementary material for: Choline kinase alpha impairment overcomes TRAIL resistance in ovarian cancer cells
Source: J Exp Clin Cancer Res. 2021 Jan 4;40:5. doi: 10.1186/s13046-020-01794-6 (PMC7780408; doi:10.1186/s13046-020-01794-6)
Supplement: Supplementary file 1 — Additional file 1: Supplementary table 1 Full list of antibodies, probes and siRNA used. [file 13046_2020_1794_MOESM1_ESM.pdf]

SUPPLEMENTARY TABLE 1

Full list of antibodies, probes and siRNA used

Antibodies for Western blotting		
Target	***Source***	***Product code***
ChoKα	Sigma-Aldrich	HPA024153
TRAIL-R2	Sigma-Aldrich	HPA023625
β-actin	Sigma-Aldrich	A2066
Vinculin	Cell Signalling	4650S
PARP	Cell Signalling	46D11
Caspase-3	Cell Signalling	9662
Bcl-2	Santa Cruz	sc-509
Bid	Cell Signalling	2002
XIAP	Santa Cruz	sc-55550
Cav-1	Santa Cruz	sc-894
Flot-1	Cell Signalling	3253S
Rabbit IgG-HRP	Sigma-Aldrich	GENA934
Mouse IgG-HRP	Sigma-Aldrich	GENA931
Antibodies for flow cytometry		
Target	***Source***	***Product code***
TRAIL-R2	R&D System	FAB6311P
TRAIL-R1	R&D System	FAB347P
TRAIL-R3	Sigma-Aldrich	PRS2299
TRAIL-R4	Sigma-Aldrich	PRS2021
Mouse IgG1-PE	Miltenyi Biotec	130-092-212
Mouse IgG2a-PE	Miltenyi Biotec	130-091-835
Rabbit IgG-ALEXA 488	Thermo Fisher	A11034
Annexin V-FITC	Abcam	ab14085
Probes for quantitative Real-Time PCR		
Target	***Source***	***Product code***
CHKA	Thermo Fisher	HS00608045_m1
CHKB	Thermo Fisher	HS01925200_s1
TNFRSF10B	Thermo Fisher	HS00366278_m1
TNFRSF10A	Thermo Fisher	HS00269492_m1
TNFRSF10C	Thermo Fisher	HS00182570_m1
TNFRSF10D	Thermo Fisher	HS00388742_m1
RPL13A	Thermo Fisher	H201926559_g1
GAPDH	Thermo Fisher	HS03929097_g1
small interfering RNA		
Target	***Source***	***Product code***
CHKA NM_212469	Dharmacon	siGENOME smart pool
CHKA NM_001277	Dharmacon	
CHKB NM_001277	Dharmacon	
non-targeting no.2	Dharmacon	
