# Supplementary material for: Choline kinase alpha impairment overcomes TRAIL resistance in ovarian cancer cells
Source: J Exp Clin Cancer Res. 2021 Jan 4;40:5. doi: 10.1186/s13046-020-01794-6 (PMC7780408; doi:10.1186/s13046-020-01794-6)
Supplement: Supplementary file 2 — Additional file 2: Supplementary Figures 1-5. [file 13046_2020_1794_MOESM2_ESM.pdf]

SUPPLEMENTARY FIGURES

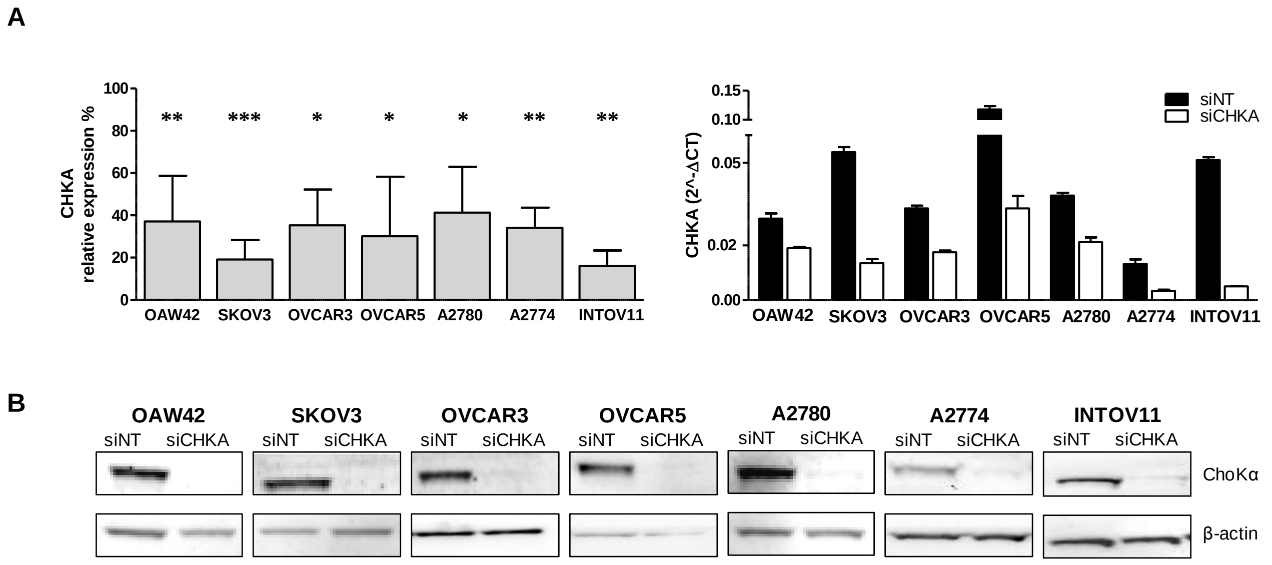

Fig. S1 - Transient siCHKA efficiently reduced the relevant transcript and protein expression. A. Relative quantification of *CHKA* transcript expression reported as percentage of 2^-ΔCT siCHKA/siNT ratio (*left panel*), asterisks are referred to statistically significant differences; a representative experiment is shown (*right panel*). **B.** Representative Western blotting analysis for ChoKα protein expression in OC cell lines following 72 h transient *CHKA* silencing. β-actin was used as loading control.

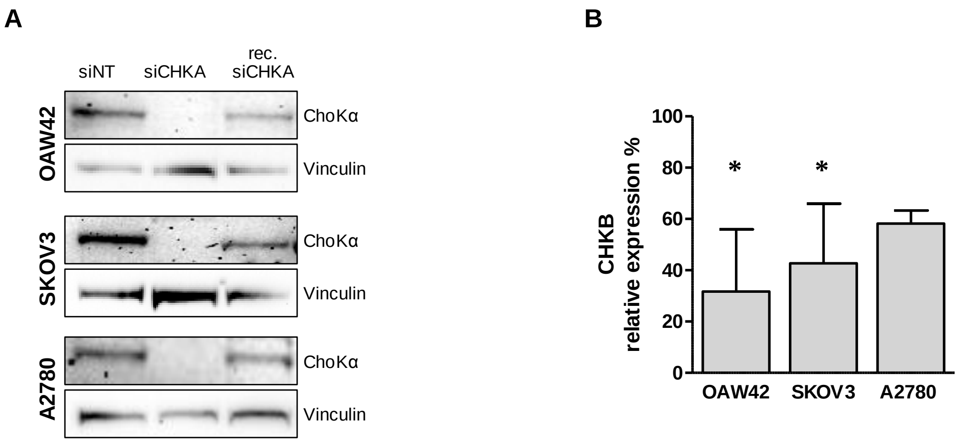

Fig. S2 - OC cells' sensitivity to sTRAIL treatment is specifically dependent upon *CHKA* downmodulation.

A. Representative Western blotting analysis for ChoKα protein expression in OC cell lines following *CHKA* transient silencing and recovery of ChoKα expression. Vinculin was used as loading control. **B.** Relative quantification of *CHKB* transcript expression reported as percentage of 2^-ΔCT siCHKB/siNT ratio, asterisks are referred to statistically significant differences.

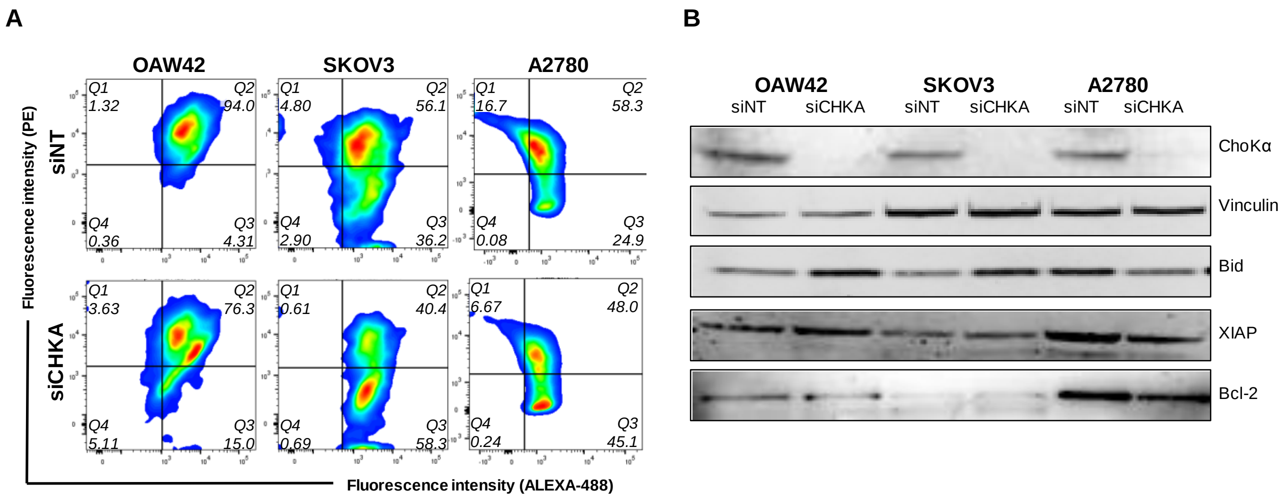

Fig. S3 - *CHKA* silencing affected mitochondrial membrane potential but did not modify expression of common pro/anti apoptosis molecules.

A. Representative flow cytometry analysis of mitochondrial membrane potential (ψ_m) in siCHKA and siNT cell lines as detected by JC-1 labeling. A decrease in the red/green fluorescence intensity ratio indicates mitochondrial membrane depolarization (for each quadrant, the relative cell percentage on total count events is reported). **B.** Representative Western blotting analysis for Bid, XIAP and Bcl-2 protein expression in OC cell lines following transient *CHKA* silencing. Vinculin was used as loading control.

A

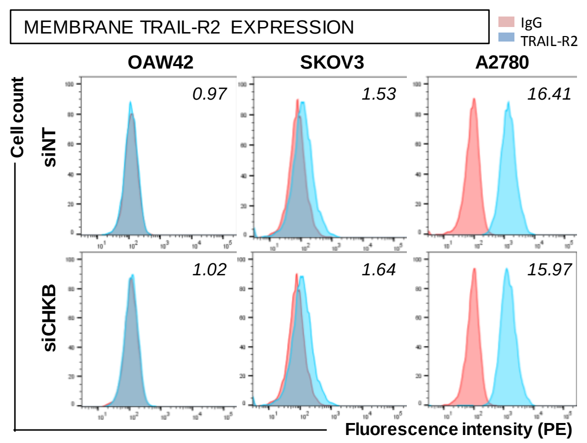

B

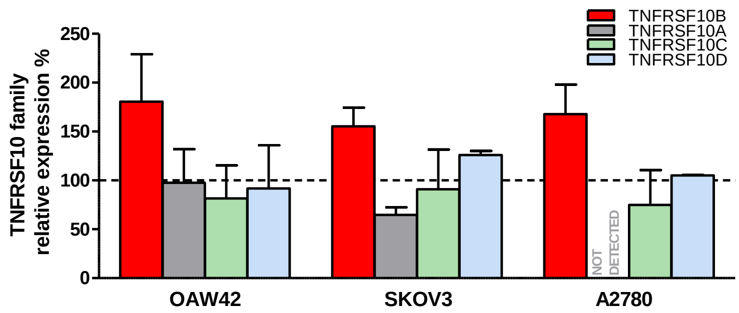

C

Fluorescence index ratio 4°C		siNT	siCHKA
TRAIL-R1	OAW42	1,14	1,08
	SKOV3	0,97	0,90
	A2780	1,03	0,96
TRAIL-R3	OAW42	1,17	1,36
	SKOV3	1,01	1,01
	A2780	1,08	1,10
TRAIL-R4	OAW42	1,76	1,85
	SKOV3	1,10	1,06
	A2780	1,05	1,06

D

Fluorescence index ratio 37°C		siNT	siCHKA
TRAIL-R1	OAW42	1,26	1,29
	SKOV3	1,35	1,07
	A2780	1,03	1,06
TRAIL-R3	OAW42	1,33	1,45
	SKOV3	1,09	1,17
	A2780	0,98	1,04
TRAIL-R4	OAW42	1,37	0,96
	SKOV3	0,97	1,16
	A2780	1,16	1,19

Fig. S4 - TRAIL-R2 is the only TRAIL-Rs family member specifically affected by siCHKA. **A.** siCHKB does not affect TRAIL-R2 membrane expression as detected by flow cytometry analysis. Fluorescence index are reported **B.** *TNSFRSF10A, TNFRSF10C, TNSFRSF10D* did not significantly increase in siCHKA cells compared to control. Relative quantification of transcripts expression are reported as percentage of 2^-ΔCT siCHKA/siNT ratio. *TNFRSF10B* expression (red bar) already reported in Fig. 4 is shown for comparison. **C, D.** Representative fluorescence indexes detected by flow cytometry summarizing membrane surface expression of TRAIL-Rs in siCHKA and control OC cell lines detected following primary antibodies incubation at 4°C or 37°C, respectively.

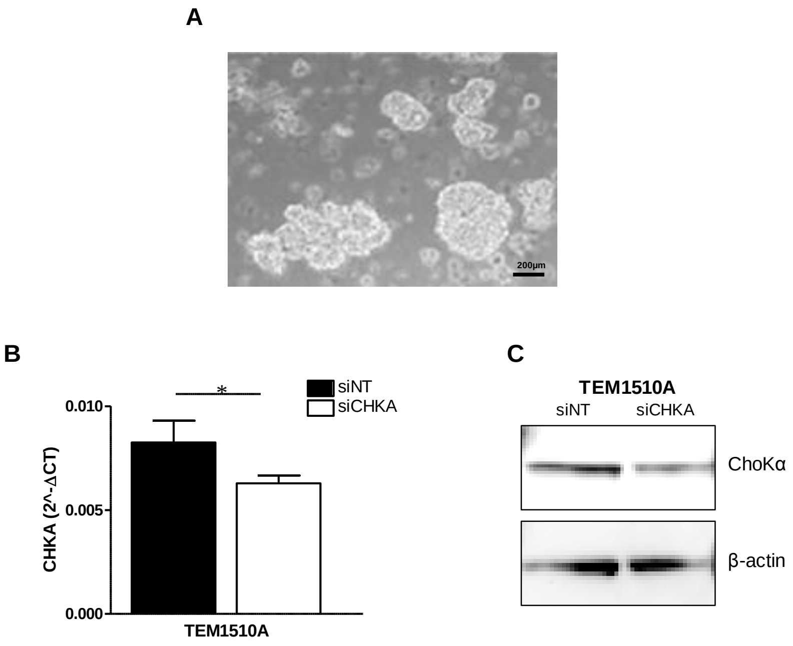

Fig. S5 - Assessment of siCHKA efficacy on *ex vivo* OC cells collected from patients' ascites. **A.** Live 20× microscope image of typical appearance of *ex vivo* OC cells in patient's ascitic fluid. **B.** Relative quantification of *CHKA* transcript expression in siCHKA OC cells derived from ascites (patient TEM1510), asterisks are referred to statistically significant differences. **C.** Representative Western blotting analysis for ChoKα protein expression in *ex vivo* OC cells following transient *CHKA* silencing. β-actin was used as loading control.
